# MicroRNAs as the Sentinels of Redox and Hypertrophic Signalling

**DOI:** 10.3390/ijms232314716

**Published:** 2022-11-25

**Authors:** Filip Kolodziej, Brian McDonagh, Nicole Burns, Katarzyna Goljanek-Whysall

**Affiliations:** 1 Department of Physiology, School of Medicine, CMNHS, University of Galway, H91TK33 Galway, Ireland; 2 Institute of Life Course and Medical Science, University of Liverpool, Liverpool L69 3BX, UK

**Keywords:** microRNAs, muscle, redox, sarcopenia, ageing

## Abstract

Oxidative stress and inflammation are associated with skeletal muscle function decline with ageing or disease or inadequate exercise and/or poor diet. Paradoxically, reactive oxygen species and inflammatory cytokines are key for mounting the muscular and systemic adaptive responses to endurance and resistance exercise. Both ageing and lifestyle-related metabolic dysfunction are strongly linked to exercise redox and hypertrophic insensitivity. The adaptive inability and consequent exercise intolerance may discourage people from physical training resulting in a vicious cycle of under-exercising, energy surplus, chronic mitochondrial stress, accelerated functional decline and increased susceptibility to serious diseases. Skeletal muscles are malleable and dynamic organs, rewiring their metabolism depending on the metabolic or mechanical stress resulting in a specific phenotype. Endogenous RNA silencing molecules, microRNAs, are regulators of these metabolic/phenotypic shifts in skeletal muscles. Skeletal muscle microRNA profiles at baseline and in response to exercise have been observed to differ between adult and older people, as well as trained vs. sedentary individuals. Likewise, the circulating microRNA blueprint varies based on age and training status. Therefore, microRNAs emerge as key regulators of metabolic health/capacity and hormetic adaptability. In this narrative review, we summarise the literature exploring the links between microRNAs and skeletal muscle, as well as systemic adaptation to exercise. We expand a mathematical model of microRNA burst during adaptation to exercise through supporting data from the literature. We describe a potential link between the microRNA-dependent regulation of redox-signalling sensitivity and the ability to mount a hypertrophic response to exercise or nutritional cues. We propose a hypothetical model of endurance exercise-induced microRNA “memory cloud” responsible for establishing a landscape conducive to aerobic as well as anabolic adaptation. We suggest that regular aerobic exercise, complimented by a healthy diet, in addition to promoting mitochondrial health and hypertrophic/insulin sensitivity, may also suppress the glycolytic phenotype and mTOR signalling through miRNAs which in turn promote systemic metabolic health.

## 1. Introduction

### 1.1. Challenging the Oxidative Stress Theory of Muscular Function Decline

Maximal aerobic capacity (VO_2_max) is the maximum volume of oxygen that can be delivered to and consumed by continuously working muscles during a maximal graded exercise test (GXT) [1,2,3]. The main factors contributing to VO_2_max are the cardiac output, blood volume, haemoglobin concentration-oxygen carrying capacity. These features determine the O_2_-carrying capacity. At the molecular level, VO_2_max is dependent on skeletal muscle mitochondrial density and metabolic enzyme capacity, which determine the O_2_-extracting capacity [4,5]. VO_2_max is a strong predictor of both endurance exercise performance [3,4] and increased risk of all-cause mortality [6]. Both the cardiovascular and skeletal muscle components of VO_2_max deteriorate with age [7,8] in addition to reduced or complete cessation in training volume and/or intensity [9]. The functional declines are coupled with reduced mitochondrial oxidative capacity due to the accumulation of dysfunctional mitochondria, elevated production of reactive oxygen species (ROS), consequent damage of the mitochondrial DNA (mtDNA) and decreased protein content and quality in striated muscles [10,11]. Vast evidence points toward mitochondrial dysfunction as the instigating defect for age-related muscle loss (sarcopenia) [12].

Paradoxically, the production of endogenous ROS during exercise is necessary for optimal metabolic and contractile function, as well as adaptive signalling [13,14]. Mitochondrial ROS have been reported to orchestrate muscle cell regeneration following a mechanical injury [15]. An adequate reduction-oxidation (redox) balance is critical for the maintenance of optimal muscle mass, as ROS overload originating from mitochondria and/or cytosolic oxidases, modulates protein/organelle degradation mechanisms including the proteases, autophagic-lysosomal, and proteasome systems [16]. A disrupted redox environment is deemed a culprit or secondary contributor to a plethora of metabolic and age-related pathologies such as atherosclerosis, hypertension, obesity, congestive heart failure (CHF), Alzheimer’s disease (AD) and type 2 diabetes mellitus (T2DM) [17,18,19]. On the contrary, a pro-oxidant redox disturbance of correct magnitude and frequency is recognised as oxidative eustress [20]. Oxidative eustress elicited by exercise provides the robustness and functionality of muscular tissue by facilitating the activation of the transcriptional factors, nuclear factor erythroid-derived 2-like 2 (Nrf2), a “master regulator of antioxidant response”, and peroxisome proliferator-activated receptor (PPAR)-γ coactivator-1α (PGC-1α), a “master regulator of mitochondrial biogenesis” [21,22,23]. However, there is no consensus whether disrupted redox homeostasis contributes to the fast-twitch dominant age-related muscle atrophy [11]. Sarcopenic mitochondrial dynamics are characterised by impairment of both mitochondrial fusion and fission, resulting in the accumulation of dysfunctional mitochondria, as well as mitophagy and mitochondrial biogenesis. Transgenic mice lacking both mitochondrial fusion (dynamin-like 120 kDa protein; Opa1) and fission (dynamin-related protein 1; Drp1) proteins closely resembled the sarcopenic mitochondrial dynamics, but also exhibited decreased secretion of senescence factors, oxidative stress, denervation, and inflammation, despite a significant muscle weakness [24].

Multiple human and animal-based studies suggest that maintenance of mitochondrial quality control through exercise, nutritional or pharmacological interventions slows down the progression of sarcopenia and rate of functional decline [25]. Moreover, six weeks of isolated knee-extension endurance training enhanced the activity of the mitochondrial enzymes (citrate synthase, mitochondrial complexes I–IV) in the quadriceps muscle of young men independent of the mitochondrial volume and mtDNA content [26]. Conversely, four weeks of deconditioning resulted in a similar magnitude of decline in enzymatic activity, also without any significant alterations to mitochondrial dynamics or mtDNA content [26]. This suggests that the changes in transcription or translatory capacity/output are key determinants of muscle plasticity in both enhancement and decline of muscle function. Several studies showed that cardiovascular and neuromuscular function, as well as skeletal muscle mitochondrial efficiency, followed by oxidative capacity can be significantly improved with several weeks of endurance training in previously inactive individuals [27,28,29]. Similarly, prolonged resistance training may result in increased muscle strength and hypertrophy [30,31,32]. However, the degree of mitochondrial [33,34] and hypertrophic [31,35] adaptability is somewhat reduced with ageing. Deconditioning in older adults results in a more pronounced rate of loss of the mitochondrial and hypertrophic adaptations [36,37].

Physiological and anatomical adaptations are believed to occur due to molecular signalling cascades resulting in “mRNA bursts”, increased transcription of specific target genes and subsequent fine-tuning of the translation into proteins which convey the enhanced phenotype [38,39]. Recent studies suggest that the activation of the translatory machinery (phosphorylation of eukaryotic initiation factor 4E binding protein 1 (4E-BP1) and the 70-kDa ribosomal protein S6 kinase (S6K1)) and ribosomal biogenesis coupled with protein synthesis in response to a bout of resistance exercise are significantly reduced in elderly vs. young men [40,41]. Muscles from older individuals also exhibit an impaired differentiation of progenitor cells (myoblasts) into lineage-committed myocytes, strongly correlating to attenuated endoplasmic reticulum (ER) response/protein folding post-exercise [42]. However, the mRNA and protein levels of the differentiation-promoting myogenesis regulating factor (MRF), myogenic differentiation 1 (MyoD1) are significantly higher in older people at rest and post-exercise [43,44,45], suggesting a failed compensation. The translation of all MRFs, including MyoD1, is tightly regulated by multiple mechanisms, including the endogenous mRNA-silencing molecules, the microRNAs (miRNAs) [46,47]. miRNAs are short, approximately 22 nucleotides long, single-stranded RNAs. Each miRNA family has a unique “seed-sequence” located at the 5′ end, which allows partial binding to a target site usually within the 3′ untranslated region (3′-UTR) of the target mRNA [48,49]. miRNAs play an instrumental role in muscle development and growth [46,47,50], mitochondrial remodelling [51,52,53] and protein quality control including unfolded protein response (UPR) [54]. Therefore, characterising the links between the deterioration of muscle mass, mitochondrial function, redox homeostasis and miRNA expression with ageing or detraining may be key to deciphering the extent to which disrupted redox signalling contributes to the process of muscular decline.

### 1.2. miRNA Memory Cloud Perpetuates the Exercise-Induced Adaptive Phenotype

miRNA levels are mainly dictated by their rate of synthesis and degradation. There is limited evidence on the regulation of the miRNA processing in response to an exercise bout and exercise training compared to miRNA expression. A significant increase in miRNA biosynthetic enzymes: Drosha, Exportin 5 and Dicer mRNA, 3 h following a single bout of 60 min continuous stationary cycling at 70% of pre-determined VO_2_peak was reported in young untrained men [55]. However, in the same study, the resting mRNA levels of Drosha, Exportin 5 and Dicer after a 10-day intervention including five sessions of moderate-intensity continuous training (MCT) and four of high-intensity interval training (HIIT) returned to the pre-training values [55]. This suggests that the transcriptional activation of miRNA biogenesis in response to exercise may be transient. miRNAs downregulated at the post-intervention time-point were negatively correlated with histone deacetylase 4 (HDAC4) mRNA, which was elevated 19-fold acutely and only 4-fold at the end of the intervention [55]. HDAC-dependent histone deacetylation results in a more condensed DNA conformation and decreased space for transcriptional complexes to induce their target genes [56]. Aerobic exercise-induced signalling through adenosine monophosphate-activated kinase (AMPK) and calcium/calmodulin-dependent kinase (CaMK) promote translocation of HDAC4 from the nucleus to cytoplasm, releasing the repression of HDAC4 target genes such as myocyte enhancer factor-2C (MEF-2C), glucose transporter 4 (GLUT4), myosin heavy chain (MyHC), PGC-1α, heat shock cognate 71 kDa protein (Hsc70), and paired box family transcription factor 7 (PAX7) [57]. Therefore, regulation of HDAC4 expression and subcellular localisation may play a key role in muscle adaptation and plasticity. Of note, HDAC4 is upregulated in muscle atrophy due to denervation, immobilisation or ageing [58,59,60]. Therefore, the counterintuitive increase in HDAC4 mRNA in sedentary participants (all less than 2 h of exercise a week) who suddenly performed nine moderate-heavy sessions in a space of 10 days [55], may suggest that over-reaching/unaccustomed training load might promote a skeletal muscle epigenetic landscape unfavourable for adaptation and regeneration similar to atrophic conditions [61]. On the other hand, lower levels of HDAC4 in muscle-specific knock out mice resulted in improved muscle regeneration following nerve injury due to de-repression of the fibroblastic growth factor (FGF) binding protein-1 (FGFBP-1) [62] suggesting a potentially pleiotropic role of HDAC4.

Conditional deletion of DICER in skeletal muscles resulted in impaired regeneration in response to injury [63] but did not affect the protein expression and enhancement of maximal oxidative capacity in isolated fibres following endurance exercise training in mice [64]. Therefore, immediate miRNA biogenesis appears more important for promoting adaptation and/or recovery from damaging modalities such as heavy weightlifting, unaccustomed eccentric loading or unusually intense/prolonged endurance exercise [65]. However, both studies reported only a partial reduction in miRNA expression following several weeks of inhibiting Dicer [63,64]. Therefore, based on current evidence determining if miRNA response is mandatory for mounting exercise-induced muscle adaptation is unclear. A recent study by Margolis explored the miRNA blueprint induced by exercise over time [66,67]. Specifically, 3 days of military training in energy deficit was shown to increase skeletal muscle mRNA levels of rate-limiting fatty-acid transporter carnitine-palmitoyl transferase A (CPT1A) more than in energy balance, despite that expression of its upstream transcriptional factor, peroxisome proliferator-activated receptor-α (PPAR-α) was not significantly different between the two groups [66]. Notably, miR-34a-5p, a negative regulator of PPAR-α was significantly more abundant in the negative energy balance group, suggesting that other compensatory mechanisms maintained the elevated levels of CPT1A transcripts despite the apparent suppression of the key activator. Analogously, muscle protein synthesis rates were reported to remain elevated in chronically exercised rats, despite a significant fall in the activation of the “anabolic master switch” mechanistic target of rapamycin (mTOR) with subsequent exercise sessions [68]. Therefore, it is plausible that exercise-induced changes in the skeletal muscle miRNA blueprint downregulate the acute effectors of exercise-stress response, but simultaneously allow “more efficient” transcription and subsequently translation owing to favourable miRNA-induced epigenetic cloud [67], with miRNA cloud concept, similar to feedback or feedforward loops, suggesting that a set of microRNAs is regulated following a specific stimulus that can convey or perpetuate the response to a stimulus such as exercise over time. Moreover, significantly higher pre-miRNA pools were reported in older relative to younger adults suggesting the impairment of exercise-triggered miRNA processing with age and sedentary lifestyle [69]. Thus, potentially contributing to the differential miRNA signature and reduced hypertrophic and translatory adaptability in old compared to young [31,35,40,41].

### 1.3. Role of miRNAs in Myogenesis—The Orchestration of the Metabolic Symphony

The exact involvement of satellite/muscle stem cells (MuSCs) and myogenesis in adaptation to endurance exercise remain undefined [70]. Despite contradictory evidence, recent experiments utilising timed in vivo myonuclear labelling suggest that satellite cells play an important role in training-induced hypertrophy [71]. In addition to participation in acute response through myonuclear accretion, MuSCs were suggested to contribute to synthesis of ribosomal proteins and satellite cell-derived myonuclei were shown to have altered methylation status that favours cell-to-cell signalling, possibly “rejuvenating” the myofibers and promoting long-term hypertrophy [71,72,73]. It also appears that MuSC miR-1 expression reflects the epigenetic memory of previous training, as it enhances ongoing growth and likely facilitates re-growth after a period of detraining in mice [74].

miRNAs play key roles in myogenic development and regeneration from injury. Dicer KO mouse embryos failed to thrive, while the conditional muscle-specific KO resulted in profound muscle hypoplasia and delayed muscle development [50,75]. Marked shifts in the expression of miRNAs including muscle-specific myomiRs occur during the transitions between the stages of myogenesis [76,77]. The transcription of myomiRs is regulated by the MRFs including the MyoD1, MYF5 (myogenic factor 5), MYOG (myogenin) and MYF6 (myogenic factor 6), as well as MEF-2C [78,79]. Together, with the paired box family transcription factors PAX3 and PAX7, a timely transcriptional activity of the MRFs is crucial for orderly myogenesis during embryogenic development and regeneration [80,81]. Consistently, miRNAs also exhibit a time-dependent expression pattern throughout the myogenic programme. Progression of myogenesis depends on timely shifts between glycolytic and mitochondrial energy metabolism [82,83,84] and mitochondrial dynamics [85,86,87].

miRNAs promote MuSC commitment by regulating Pax7 expression [88,89,90,91] and de-repressing of MEF-2C by targeting HDAC4 [92]. Of note, miRNAs initiate the initial myoblast differentiation in vitro by targeting the uncoupling protein 2 (UCP2) [93]. UCP2 is a chemiosmotic decoupler, protecting from proton leak and ROS generation [94]. This highlights the link between miRNAs and redox balance for adequate muscle growth/regeneration. Differentiation into myocytes is also promoted by miRNA-dependent silencing of phosphatase and tensin homolog (PTEN) and forkhead box protein O1a (FOXO1a) [95], the downstream effectors of the phosphoinositide-3 kinase/protein kinase B (PI3K/Akt) pathway [96]. IGF-1-dependent activation of the PI3K/Akt/mTOR pathway has been strongly linked to anaerobic/glycolytic phenotype, myoblast proliferation and muscle hypertrophy [97]. This again suggests that miRNAs modulate the progression of the myogenic program by regulating the metabolism-regulating signalling pathways. Thus, miRNA-dependent post-transcriptional regulation emerges as the bridge connecting muscle metabolic phenotype with skeletal muscle development, regeneration and hypertrophy.

### 1.4. The Role of miRNA Networks in Muscle and Metabolic Health—The Context Matters as Much as the Content

miRNAs regulate muscle fibre-type and metabolic performance [98,99] and muscle plasticity/adaptability to imposed stressors. miR-208a, miR-208b and miR-499 form a loop responsible for the maintenance of the slow-twitch skeletal muscle fibres during inactivity and protection from fast-twitch hypertrophy in the overloaded heart [98,100]. Furthermore, miR-499 promotes PGC-1α activation in skeletal muscle by targeting the AMPK suppressor, folliculin-interacting protein 1 (Fnip1) [101]. AMPK is well known to oppose pro-glycolytic mTOR signalling through several pathways [102,103]. Hence, miRNAs cross-regulate the antagonistic pathways and their resultant phenotypes.

miR-378a-5p and its passenger strand, miR-378a-3p are encoded within the first intron of PPAR γ coactivator-1β (PGC-1β) [104]. The global deletion of both miR-378 strands in mice promoted their resistance to HFD-induced obesity, by improving hepatic fatty-acid oxidation [104]. Paradoxically, miR-378-3p appears to mediate the anti-diabetic properties of AMPK-activating metformin in muscle cells exposed to hyperglycaemic stress, by promoting the expression of the mitochondrial transcription factor A (TFAM) and enhanced mitophagy [105]. Analogously, metformin is a well-known anti-diabetic drug restoring insulin sensitivity [106]. However, middle-aged to older adults at risk of or suffering from metabolic syndrome who undertook an exercise intervention in combination with metformin therapy exhibited a reduced VO_2_max increase [107,108]. Thus, the contrasting effects of miR-378 KO suggests that miRNA function may depend on the context of the tissue it is expressed in and the systemic stressors, such as exercise.

miR-378-3p also favoured a pro-oxidative and suppressed hypertrophic/glycolytic phenotype in the myoblasts [109], cardiomyocytes [110], and adipocytes [111]. Consistently, miR-378 was shown to prompt myogenic differentiation by targeting the HDAC4 [112] and BMP4 [113], both of which play an integral part in setting the proliferative/glycolytic muscle cell identity [114,115]. HDAC4 suppresses the slow-twitch oxidative fibre development through deacetylation of the MEF-2C [116] as well as PGC-1α and MyHC genes [117]. Considering that in vitro treatment of myotubes with tumour necrosis factor α (TNF-α) or interferon γ (IFN-γ) resulted in decreased expression of miR-378 [109], an inflammatory/diseased state could impair the miRNA-dependent orchestration of muscle and systemic metabolism.

**Figure 1:**
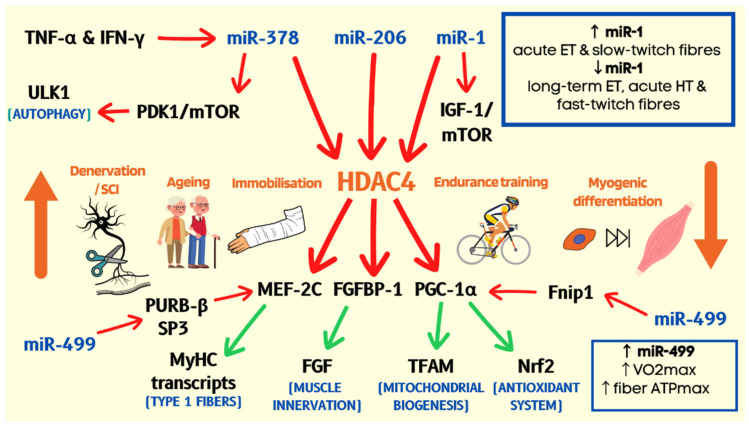
Muscle miRNAs regulate muscle oxidative/slow twitch phenotype and mass. HDAC4 is upregulated in pro-atrophic conditions [58,59,60], inhibiting the expression of MEF-2C and PGC-1α [116,117]. This is associated with decreased transcription of muscle-contractile, mitochondrial and antioxidant proteins. HDAC4 also suppresses the expression of FGFBP-1, reducing the binding and activation of neurogenic FGFs [62]. miR-206 is upregulated following acute high-intensity exercise [47], contributing to increased muscle innervation. HDAC4 is targeted by miR-378, which promotes autophagy by releasing PDK1/mTOR-dependent suppression of autophagy-inducing ULK1 [109,112]. miR-378 levels are diminished by pro-inflammatory cytokines, TNF-α and IFN-γ [109]. HDAC4 nuclear abundance/activity decreases in response to endurance exercise [57] and in differentiating muscle progenitors [112]. miR-1 blocks HDAC4 expression and is elevated in slow-twitch relative to fast-twitch fibres. miR-1 is increased following acute endurance training (ET) compared to long-term (ET) and decreased after acute hypertrophy training [47]. Thus, ET may acutely promote miRNA profile permissive to the enhancement of oxidative phenotype while maintaining muscle protein synthesis and proliferative potential long-term. miR-499 opposes the effects of HDAC4 and strongly correlates with aerobic and metabolic capacity [121]. Red arrow—inhibition; green arrow—upregulation.

HDAC4 expression is upregulated in skeletal muscles in multiple atrophy-favouring conditions such as denervation, immobilisation or ageing [58,59,60]. Remarkably, mice injected with an HDAC4 inhibitor into their gastrocnemius muscle were resistant to denervation-induced atrophy, as their PGC-1α expression and oxidative capacity were preserved [117]. This finding is consistent with endurance exercise-induced AMPK and CaMK reducing HDAC4 in nuclear domain [57], however muscle-specific HDAC4 KO resulted in more rapid regeneration of muscle following nerve injury [62]. Moreover, other MEF-2C repressors, PURB-β and SP3, targeted by miR-208b and miR-499, were shown to be associated with the inactivity-induced muscle atrophy [118]. Both miR-208b and miR-499 are downregulated in skeletal muscles following a prolonged hindlimb suspension in mice [119], and spinal cord injury in humans [120]. Of note, the expression of miR-208b and miR-499, as well as the critical pro-oxidative factor, oestrogen-related receptor-γ (ERR-γ), strongly correlated with the enhanced oxidative capacity (fibre ATPmax, and VO_2_max) and the slow-twitch fibre content in physically active and sedentary adults [121]. Moreover, miR-1 is increased following acute endurance training (ET) compared to long-term (ET) and decreased after acute hypertrophy training [47]. Therefore, muscle miRNA-dependent re-configuration of the epigenetic histone modifications and consequent regulation of oxidative metabolism and phenotype might contribute to the decline in muscle aerobic function and mass with ageing, detraining or declined physical activity (Figure 1).

The function of each miRNA is not only dependent on the context of the tissue it is operating in but also the functionality of the “network-companion” miRNAs. miR-499 and miR-378 corresponding targets Fnip1 and HDAC4, suppress the activation of the heat shock protein 90 (Hsp90) and Hsc70, respectively [117,122]. Heat shock proteins are molecular chaperones necessary for the cellular stress response, including the recognition of damaged/misfolded proteins [123]. Therefore, dysregulation of the miRNA networks may result in the inaccurate balancing of the signalling pathways as well as inadequate protein quality control, compromising muscle development and maintenance [124] including the metabolic, oxidative and mechanical stress response [125,126,127]. Hence, recognising the differences in the miRNA network in adaptive stress response (e.g., exercise) vs. maladaptive (e.g., disease) can unveil the mechanisms behind muscular decline with detraining or ageing.

### 1.5. Muscle miRNA Networks in Metabolic Disease, Ageing and Exercise Training—Fine-Tuning Thermostat of Redox and Anabolic Sensitivity

Mitochondrial quality control plays a prominent role in sarcopenia [16,24,25]. Abnormally swollen mitochondria were observed in skeletal muscles of old mice despite the upregulated expression of autophagic proteins including p62, PARK-2, and DJ-1 suggesting impaired autophagy [53]. The apparent mitophagy arrest was released by treatment with miR-181a, which prevented accumulation of the above proteins and restored the mitophagic flux in vitro and in vivo. miR-181a appears to regulate both mitochondrial biogenesis and mitophagy. The effects of miR-181a on the autophagic markers in old sedentary mice were similar to those that were subjected to in situ stimulation protocol mimicking exercise. Consistently, miR-181a treatment increased peak isometric force, muscle fibre size and expression of electron transport chain proteins owing to induction of transcriptional factor TFAM [53]. TFAM expression is upregulated by the exercise-dependent Nrf2 response [23]. Exercise-induced Nrf2 transcriptional activity was shown to be essential to igniting both mitochondrial fusion and fission by regulating the stability of the mitochondrial fission protein Drp1 [128,129]. Furthermore, Nrf2 deletion in mice severely accelerated muscle atrophy, by directly disrupting mitochondrial dynamics in an age-dependent fashion [130]. Notably, inhibition of Drp1-dependent mitochondrial division markedly blunted myogenic differentiation [86]. Thus, considering the impaired Nrf2 exercise response in old rodents and elderly sedentary adults [22,131,132] progressing “hormetic impotence” could be linked to the aberrant miRNA blueprint perpetuating the sarcopenic phenotype. Correspondingly, a ROS-induced master pro-inflammatory transcriptional factor, nuclear factor of activated B-cells Kappa (NF-κB) is chronically active in ageing muscles markedly diminishing the regenerative capacity through restraining MuSC differentiation [133,134].

In addition to changes in Nrf2 activation, the induction of other redox-sensitive transcription factors such as heat shock factor 1 (HSF1), NF-ΚB and activating protein 1 (AP-1) fails in old mice following exercise [135,136,137]. Consistently, acute activation of NF-κB with exercise is minimal in obese and effectively unnoticeable in type 2 diabetic young adults [138]. Cobley and colleagues suggested that this acute transcriptional inability is due to persistent baseline Nrf2 activation, as well as elevated NF-κB levels, contributing to chronic oxidative stress noise in inactive muscles, decreasing the signal-to-noise ratio and leading to dampened exercise-elicited pro-oxidant shifts [132]. This is reflected by the baseline elevation of the antioxidant enzymes SOD2 and catalase in the muscles of the aged compared to young rodents [135,136,137]. The non-functional hyper-expression and activity of the antioxidant systems shift redox balance toward excessively reduced, resulting in “reductive stress” that hampers myogenic differentiation [139].

Of note, antioxidant supplementation significantly improves strength performance, as well as mitochondrial and metabolic adaptability in elderly men and women participating in routine combined exercise training (CXT) [140]. Therefore, reduction of the oxidant “noise” in the aged muscular milieu plausibly returns the hormetic redox sensitivity. However, a detrimental effect of long-term Vitamin C and E supplementation was observed in young adults, who experienced diminished SOD2, PGC-1α and p38 mitogen-activated protein kinase (p38 MAPK) induction by endurance training in comparison to the placebo groups [141,142,143].

Increasing sedentary behaviour with age does not appear to be fully responsible for hormetic signalling dysfunction. Men older than 55 years of age, who engaged in lifelong endurance training, were reported to preserve some but not all translational responses to an acute bout of exercise. SOD2, Hsp72 and peroxiredoxin-5 (PRX5) failed to increase from baseline, in comparison to the increase seen in subjects between 18 and 30 years old [144]. Transcriptional p38 MAPK and PGC-1α responses responsible for mitochondrial biogenesis are retained in older individuals irrespective of training status, pointing to the failure of the antioxidant/hormetic response specifically [145,146]. However, the translatory response of mitochondrial proteins tends to decline with age, suggesting a post-transcriptional block [144]. This observation is in line with the numerous studies showing reduced protein synthetic potential and quality control in aged muscle [16,24,25,31,32,33,34,35]. This in turn could be explained by the divergent muscle miRNA profile in aged men compared to young adults at baseline and post-exercise [69]. Of note, pathway analysis of 23 miRNAs differentially expressed in young vs. older men after a single bout of resistance exercise revealed that ubiquitin-mediated proteolysis, insulin, PI3K/Akt and mTOR pathways were among those strongly influenced by age, suggesting that miRNA blueprint is directly responsible for the biosynthetic failure and age-related anabolic resistance [147]. This is consistent with data published by D’Souza and colleagues who reported a strong correlation between the changes in the expression of the critical myogenic miRs -206, -208a and -499a with the phosphorylation of mTOR pathway constituents, Akt and P70S6K, in elderly men post-resistance exercise [148]. In the same study, the anabolism-permissive miRNA blueprint was enhanced by whey protein ingestion following the training bout, pointing toward the miRNAs as the key sentinels of muscle plasticity in response to stress as well as nutritional cues [148].

Several studies reported that miR-34a, miR-93, and miR-144 targeting Nrf2 are upregulated in various tissues in aged rodents [149,150,151]. Notably, Nrf2 disrupted activation has been strongly linked to the induction of cellular senescence [152]. Recently, the expression of long non-coding RNA, MALAT1, acting as a sponge (negative regulator) for miR-34a was shown to decrease in ageing muscle cells [153]. The downregulation of MALAT1 and consequently increased miR-34a availability were shown to be ROS-dependent and resulted in the induction of the major pro-inflammatory cytokine, transforming growth factor-β1 (TGF-β1) [153]. miR-34a suppresses Sirt1 [154], which in turn is involved in PGC-1α activation and Nrf2 de-repression [23]. Sirt1 is also targeted by miR-181a, which is significantly downregulated in the skeletal muscles of old mice, likely representing a failed compensatory mechanism owing to disturbed miRNA-target interaction [155]. This impairment of target binding might be due to increased RNA oxidative damage seen with ageing and metabolic disease [156,157]. Conversely, the position-specific oxidation of miRNAs can also be conducive to maladaptive post-transcriptional modulation, as observed in cardiac hypertrophy [158].

**Figure 2:**
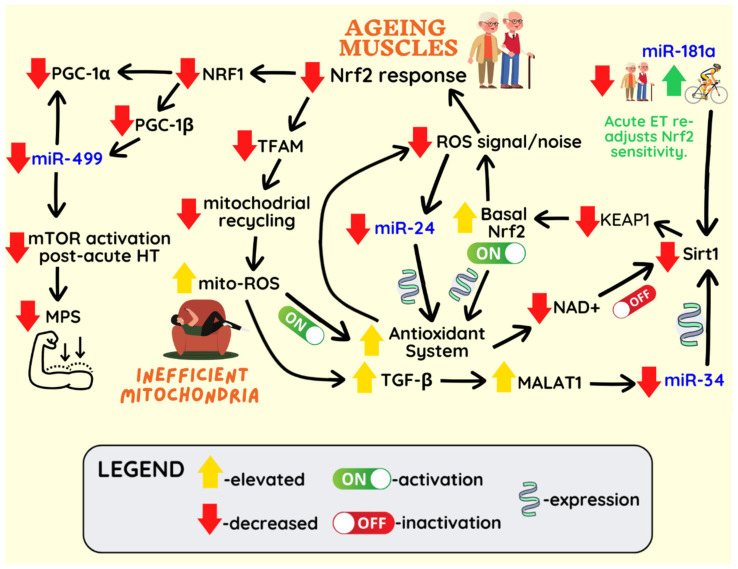
Chronic baseline ROS overload prompts miRNA-mediated pro-reductive shift, and consequent decline in redox and hypertrophic sensitivity. Nrf2 activation in response to endurance training (ET) declines in ageing muscles. This results in decreased PGC-1β, which regulates the expression of miR-499. Downregulation of miR-499 correlates with reduced mTOR phosphorylation in response to hypertrophy training (HT) in older individuals, potentially contributing to reduced muscle protein synthesis (MPS). Mitochondrial ROS production increases in older individuals or metabolically inefficient mitochondria in younger individuals. Increased ROS induces TGF-β1/MALAT1/miR-34 cascade leading to decreased Sirt1 expression. Elevated ROS levels stimulate the antioxidant systems, resulting in NAD^+^ consumption. Decreased NAD^+^ levels contribute to lower Sirt1 activation. Decreased Sirt1 deacetylase activity results in KEAP1 (negative Nrf2 regulator) downregulation. Consequently, Nrf2 nuclear translocation increases at rest, resulting in the induction of antioxidant genes (SOD2 and catalase). Hyperactivity of the antioxidant system masks the ROS bursts induced by exercise, inhibiting the expression of the ROS-induced miR-24, which normally suppresses key antioxidants (SOD1 and PRDX6). miR-181a, targeting Sirt1 is elevated in old muscles potentially to compensate for decreased Sirt1 expression. Contrastingly, miR-181a is elevated in response to a single bout (but not long-term) of high-intensity ET, possibly re-adjusting the Nrf2 breakdown rate and therefore elevating redox sensitivity via the Sirt1/KEAP1 pathway.

In addition to modulation of Nrf2 expression and electrophilic sensitivity, a great number of miRNAs including miR-24, target pro-oxidant and antioxidant enzymes [159]. miR-24 was shown to induce senescence by targeting PRDX6 in ageing myoblasts [160]. Importantly, the persistent changes to the intertwined antioxidant systems, maintained by the miRNA blueprint may result in reversible redox modifications of regulatory proteins to diverge the energy metabolism toward NADPH biosynthetic pathways, resulting in redox desensitisation [161]. Conclusively, persistent oxidant noise in metabolically inefficient/aged muscle can result in low-level chronic Nrf-2 activation and induction of particular miRNAs which in turn modulate the expression of the anti- and pro-oxidant arms of the redox system to compensate for chronic baseline ROS overload, further disrupting effective redox signalling and perpetuating the vicious cycle of hormetic and functional decline [159] (Figure 2).

### 1.6. Chronic Endurance Training-Induced miRNA Cloud and the Adaptation-Permissive Epigenetic Landscape—Revised Molecular Basis of Exercise Adaptation Model

**Figure 3:**
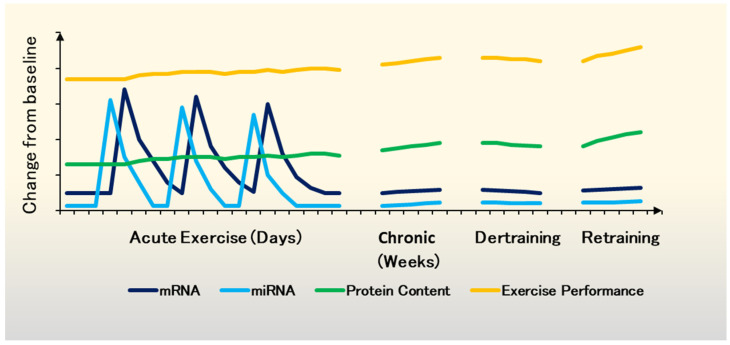
A revised model of the molecular basis of adaptation to exercise. Changes in mRNA expression (dark blue), miRNA (light blue) and protein content (green) due to acute and chronic exercise. Individual bouts of exercise are necessary for adaptation but insufficient to significantly alter the phenotype. Rapid bursts of mRNA during post-exercise recovery facilitate the expression of target genes. Alterations in mRNA expression several-fold from basal levels are typically greatest at 3–12 h after exercise and generally return to basal levels within 24 h. miRNA response follows a similar burst-like trajectory and miRNAs are thought to respond to changes in the environment quickly. The translational response is modest in comparison to mRNA and miRNAs. However, superimposing exercise bouts chronically results in the gradual accumulation of protein content contributing to enhanced exercise performance (orange). During a period of detraining, the baseline mRNA, miRNA and protein levels decrease steadily, but epigenetic changes decelerate the phenotypic decline. Initially, exercise performance does not suffer due to reduced residual fatigue (i.e., tapering). Upon return to training, people with longer exercise history achieve their previous phenotype at a faster rate than in the past or less trained individuals. This effect is likely due to the miRNA-dependent establishment or enhancement of the pro-adaptive epigenetic profile (model revised based on [39,163].

Muscle miRNA profiles vary significantly between the response to a single bout and consistent long-term endurance exercise. Muscle miR-1, miR-133a, miR-133b, and miR-206 were significantly downregulated in young adults following 2 weeks of moderate cycling training [162]. In the same study, a single 60 min bout of high-intensity cycling resulted in the increased expression of these myomiRs. However, this acute response was not present when the same protocol was repeated after the training intervention [162]. This is in line with the markers of miRNA biogenesis machinery returning to pre-training intervention values despite daily training [55] and suggests that miRNA stress-response may be a part of adaptive response and muscle memory. Notably, mathematical modelling reveals that mRNA burst trajectory is strongly coupled to microRNA trajectory, promoting effective gene expression with reduced noise [39]. Based on this evidence we propose a revised model of the molecular basis of adaptation to exercise [163] (Figure 3). The accumulation of miRNA bursts targeting histone modifying enzymes (HDACs and sirtuins) with long-term training can modulate the epigenetic landscape [61], perpetuating the initial transcriptional hormetic response into the pro-adaptive memory [66,67,74]. This epigenetic memory cloud facilitates exercise sensitivity and overload-dependent improvement of molecular and physiological status, as well as relatively swift re-acquisition of the enhanced phenotype when returning to exercise following a detraining period (Figure 3). Older adults display diminished translational response [40,41], and inferior adaptations to both aerobic and resistance exercise compared to young adults [31,33,34,35]. Consistently, the increase of 21 miRNAs in young adults in response to single bout of resistance exercise was absent in muscles of older individuals [147]. Therefore, reduced age-related signal sensitivity in exercise could be responsible for diminished miRNA burst and slower adaptation to long-term exercise as well as accelerated loss of fitness and muscle mass owing to a “stubborn” epigenetic landscape.

**Figure 4:**
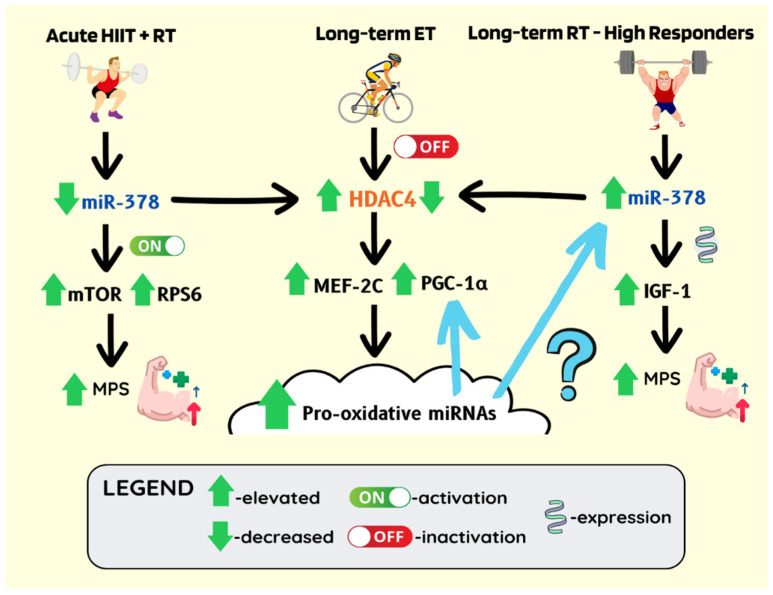
Long-term endurance training-induced miRNA cloud may facilitate oxidative and hypertrophic sensitivity (exercise memory). miR-378 is significantly higher in high responders compared to low responders following 12 weeks of hypertrophy training. Anabolic responsiveness correlates with increased IGF-1 expression. ET-induced AMPK and CaMK signalling reduces HDAC nuclear abundance. Correspondingly, miR-378 downregulates HDAC4 expression. Decreased HDAC4 epigenetic activity is permissive to the expression of muscle-specific, antioxidant, mitochondrial, and pro-oxidative miRNAs genes. The pro-oxidative miRNAs may establish a positive feedback loop maintaining the expression of the slow twitch/oxidative muscle genes and potentially be responsible for increased responsiveness/sensitivity to hypertrophic stimuli. The acute RT-dependent downregulation of miR-378 and subsequent increase in HDAC4 activity allows mounting of regenerative/inflammatory response. However, lifelong, regular aerobic training is needed to control HDAC4 activity and optimise its regulatory effects on muscle plasticity and homeostasis.

Of note, divergent miRNA profiles were observed at baseline and following resistance training in high- and low-responding (in terms of muscle hypertrophy assessed with magnetic resonance imaging) men in their early twenties [164]. Another study reported that miR-378 was increased in young high vs. low responders following a 12-week resistance program [165]. Considering miR-378 is also strongly downregulated in old compared to young men [147] and promotes glucose tolerance with improved metabolism [105,166], muscle miRNAs have the potential to serve as the predictors of susceptibility to metabolic disease and muscle atrophy at a young age. Additionally, miR-378-mediated link between glucose tolerance and anabolic responsiveness suggests that muscle metabolic efficiency (and consequently redox homeostasis) possibly maintains a miRNA cloud that is permissive to both mitochondrial and hypertrophic adaptation (Figure 4).

Of note, miR-378 supresses the expression of HDAC4 [112]. Acute HIIT combined with resistance training (RT) results in the downregulation of muscle miR-378, enhanced activation of the mTOR pathway, and consequently increased muscle protein synthesis (MPS) [167]. This consequently would increase HDAC4 expression. HDAC4 has been shown to increase at the peak of inflammatory response following muscle injury, promoting in vivo regeneration through pro-inflammatory soluble factors [168]. Skeletal muscle-specific HDAC4 KO (mHDAC4 KO) mice lose muscle structural integrity and antioxidant enzyme response following four weeks after denervation [169]. Hence, suggesting that the activation of HDAC4 activity is essential for triggering muscle regenerative and preservation responses. Similar to the sarcopenic phenotype [53], the denervated mHDAC4 KO mice fail to progress autophagy past the autophagosome formation [169]. Of note, denervated mHDAC4 KO mice treated with a lipophilic antioxidant (Trolox), methylene blue or intermittent fasting to stimulate proteasome assembly and autophagy, respectively, exhibited reduced necrosis, inflammation, and fibrosis [169]. Conclusively, the chronic upregulation of HDAC4 expression [60] and reductive shift [135,136,137] in ageing muscles may suggest a “necessary evil” allowing to preserve the less active muscles, at a price of reduced ability to transcribe muscle-specific and mitochondrial proteins [116,117], respond to redox exercise stimuli [22,131,132], timely coordinate inflammatory response and thus differentiate/mature new and grow pre-existing fibres [133,134,139]. While the consistent physical-activity and endurance exercise promoting CaMK and AMPK signalling “keep HDAC4 in check” by reducing its nuclear abundance [57]. The importance of balance between acute upregulation of HDAC4 expression with injury [168] or unaccustomed training volume/intensity [55] with fine-tune regulation of epigenetic activity [57] for maintaining muscle mass is possibly reflected in a recent study involving obese older women, where aerobic combined with resistance exercise training more effectively augmented muscle protein synthesis and myocellular quality than either intervention alone [170].

In line with the idea of stress-response specificity, a single session of resistance training has a contrasting effect on the muscle miRNA profile, compared to a single bout of aerobic exercise and appears to explain the anabolic resistance in older men due to dysregulated IGF-1 signalling [69,147,171,172]. Notably, both miR-378 strands were reportedly elevated in the plasma of obese and diabetic patients [173,174]. This observation highlights that the miRNA profile must be interpreted within the context of the investigated tissue as well as the redox and inflammatory status and the entire co-expressed miRNA network. Similarly, to maximise the potential of miRNAs as the markers of training status or trainability, they should be measured and compared against the metabolites reflective of the biosynthetic and energetic potential such as amino acids or creatine metabolites, respectively [175,176].

### 1.7. Circulating miRNAs as the Universal Language of the Hormesis Response—Biomarkers of Systemic Metabolic Health and Adaptability

The changes in miRNA blueprint in muscle atrophy depend on the context of catabolic conditions as different miRNAs had more pronounced change in starved, muscle-denervated, diabetic and cachectic mice [177]. Of note, miR-206 and miR-21 were found to be critical pro-atrophic agents in the denervated model [177]. Remarkably, silencing miRNA-206 in SOD1-G93A transgenic mice led to an HDAC4-induced reduction in the muscle secretion of FGF, affecting re-innervation process [62]. This suggests that miRNA-mediated modulation of intramuscular signalling and transcriptional activity regulates plasticity of the entire neuromuscular niche.

The effect of exercise extends beyond the neuromuscular milieu. Skeletal muscles secrete various sizes of extracellular vesicles (EVs) in the form of microparticles (MPs) or exosomes [178]. The selectively-packaged content of the muscle-derived EVs includes miRNAs, which appear to coordinate the systemic cross-talk and effective adaptation to exercise stress [179,180,181]. Vechetti and colleagues performed a gene ontology analysis of previously identified exercise-induced EV-miRNAs and found that the two mostly influenced sets of genes were involved in antioxidant response and insulin secretion [181]. Additionally, EV-miRNAs were revealed to influence immune response, protein catabolism and nervous system plasticity [178]. Electrically evoked contractile activity of sufficient intensity in one limb results in the activation of the Nrf2 response in the unstimulated limb [182]. Thus, suggesting that an unidentified “exerkine” facilitates the systemic antioxidant response. Circulating miRNAs (ci-miRNAs) emerge as attractive candidates, as EVs isolated from the aged muscle were observed to increase miR-34a content, promoting cellular senescence by inhibiting Sirt1 expression [154]. Consistently, Sirt1 has a recognised role in metabolic homeostasis and activation of the PGC-1α/Nrf2 pathway [23]. Muscle-released ci-miRNAs could potentiate the systemic effects of various hormones. Vechetti and colleagues demonstrated that muscle-derived EVs induced by mechanical overload had a permissive effect on catecholamine-stimulated white adipose tissue lipolysis via miR-1-dependent targeting of pro-lipogenic factors [183]. Additionally, Ingenuity Pathway Analysis of ci-miRNAs induced by a single bout of resistance exercise revealed that differences between young and old adults explained the magnitude of anabolic response [184]. The expression levels of six of these miRNAs were strongly correlated to the phosphorylation status of Akt and S6K1 in young vs. old adults [184]. Therefore, similarly to muscle, ci-miRNAs could act as redox and insulin sensitivity modulators at the systemic level.

Ci-miRNA responses varied significantly between the strength, endurance, and hypertrophy exercise protocols, and strongly correlated to the cytokine as well as hormonal (cortisol and testosterone) responses in young adult men [185]. However, some of the results from earlier studies on ci-miRNAs’ systemic effect have to be interpreted carefully as the responses were not specified for the free/plasma and EV-bound fractions [184,185]. Recent evidence suggests that plasma and exosomal miRNA exercise-induced changes are unrelated to one another and that of skeletal muscle in response to a single bout of MCT and HIIT in young adults [186,187]. Moreover, apart from muscles, multiple tissues including adipose, vascular endothelium and blood cells secrete EVs in response to exercise stress [188]. Hence, ci-miRNAs cannot be considered the proxies of muscular adaptations alone. Instead, vesicle-bound ci-miRNAs, in particular, could be interpreted as biomarkers of systemic health as well as redox and insulin sensitivity. Indeed, a recent study reported that exosome-derived miRNAs induced by an acute bout of endurance exercise are strongly related to the activation of IGF-1 signalling in trained vs. sedentary elderly men [189]. Furthermore, KEGG pathway analysis of exosomal miRNAs in sedentary vs. exercising young and older adults revealed a significant involvement in the signalling cascades linked to the development of diabetes, cancer, viral infections and neurodegenerative diseases [190]. Nonetheless, bearing in mind that the ci-miRNA profile changes are intensity sensitive [191], blood miRNA network responses have the potential to serve as a sophisticated marker of the systemic stress response to exercise. Therefore, ci-miRNAs may help in the future to individualise training load/intensity to optimise the systemic effects of exercise. Alternatively, considering the evidence of EVs transferred from HIIT-exercised mice enhancing glucose tolerance in sedentary mice [192], EV-miRNAs emerge as potential therapeutics for metabolic disease. Finally, there is a possibility that exercise-induced EVs derived at a young age and peak fitness can be preserved or their cargo sequenced so that they are re-administered later in life as a highly personalised “silver bullet” against the age-related muscular and systemic decline, as well as redox and insulin/anabolic resistance [193].

## 2. Conclusions

Since the discovery of miRNAs nearly three decades ago, our understanding of these intriguing molecules has grown immensely. It is now evident that miRNAs play a paramount role in regulating skeletal muscle plasticity and systemic physiology. Energy metabolism and mitochondrial dynamics are tightly coordinated with cellular functions and signalling. This principle also applies to skeletal muscles where timely rewiring of mitochondrial metabolism and mitochondrial recycling are instrumental for hypertrophy, repair and adaptation to the stress of exercise. Based on the literature to date, we suggest that miRNAs are critical in regulating the metabolism-signalling cellular symphony. Mitochondrial dysfunction and oxidative stress have been linked to disease and ageing. miRNAs may be responsible for the coordination of mitochondrial metabolism with cellular functionality and hormesis. Mitochondrial quality and function decrease with age, further accelerated by poor physical activity and diet. miRNA profiles may change to compensate for the chronic disturbance of homeostasis. However, this shift may be associated with a reduced ability for allostasis, that is change of cellular milieu in the face of hormetic challenge. This pertains to the reduced ability to sense the hormetic signals which are dampened by the ever-persisting noise of cellular stress. Enhanced mitochondrial function promotes a miRNA profile that sustains the oxidative phenotype of the skeletal muscle cells while sensitising them to anabolic stimuli. This could in part explain why mitochondria-rich oxidative muscle fibres are less prone to atrophy compared to glycolytic fibres. The limitation of this review is that it is a microRNA-centric interpretation of the literature. Future models should aim to include other global factors regulating muscle homeostasis, such as changes in transcription rate or splicing or protein modifications and stability. Our findings bring a new perspective to the exercise interference debate highlighting how endurance training may contribute to the hypertrophic adaptation. We conclude that regular endurance training suppresses the glycolytic phenotype and mTOR signalling through pro-oxidative miRNAs (miR-1, miR-378, miR-499). However, the insulin/IGF-1 signalling sensitivity is maximised owing to redox balance and optimum oxidation status of redox sensitive proteins. This potentially explains why endurance-trained athletes can maintain muscle mass despite the alleged interference. Finally, our findings emphasise the importance of regular aerobic exercise and a healthy diet for minimising the risk of sarcopenia as well as the development of systemic diseases.

## Data Availability

Not applicable.
